# An integration of bacterial probiotic extract and green nanoparticles as a safe approach for conservation of historical manuscript against fungal deterioration: an applied study

**DOI:** 10.1038/s41598-026-55481-1

**Published:** 2026-06-06

**Authors:** Mahmoud Abdel-Nasser, Amr Fouda, Gomaa Abdel-Maksoud

**Affiliations:** 1 Department of Manuscripts Conservation, Al-Azhar Al-Sharif Library, Cairo, 11511 Egypt; 2 Department of Botany and Microbiology, Faculty of Science, Al-Azhar University, Nasr City, Cairo, 11884 Egypt; 3 Heritage Science Program, School of Humanities, Faculty of International Business and Humanities, Egypt-Japan University of Science and Technology (E-JUST), New Borg El-Arab City, Alexandria, 21934 Egypt; 4 Organic Materials Conservation Department, Faculty of Archaeology, Cairo University, Giza, 12613 Egypt

**Keywords:** Historical paper conservation, *Lactobacillus plantarum*, Green TiO_2_-NPs, Analytical techniques, Biochemistry, Biological techniques, Biotechnology, Microbiology

## Abstract

This study focuses on integrations of an eco-friendly, cost effectiveness, and safe approach for conservation of historical manuscripts to avoid the risks from utilizing traditional conservation methods and synthetic fungicides, which pose dangerous effects on environment, human health, and materials of historical museums. Therefore, the deterioration aspects of an 11th-century historical manuscript, including its paper and leather binding, were assessed in relation to the influence of the corresponding fungal communities. Photographic documentation, scanning electron microscopy (SEM), X-ray diffraction (XRD), attenuated total reflectance Fourier-transform infrared (ATR-FTIR), color changes, and pH values in comparison to a control sample were used to examine the deterioration aspects. Dust, dirt, corrosion, stains, weakness, missing parts, decreasing paper crystal, changing cellulosic band wavenumbers, color change, and acidity were identified as the most degrading elements. Thirteen fungal strains were isolated from the historical manuscript and identified morphologically and molecularly as *Aspergillus flavus* (five isolates), *A. chinensis* (one isolate), *Penicillium chrysogenum* (one isolate), *Cladosporium velox* (one isolate), *Paecilomyces variotii* (two isolates), *Paecilomyces brunneolus* (one isolate), *Curvularia tamilnaduensis* (one isolate), and *Curvularia geniculate* (one isolate). These strains showed high activity for producing different hydrolytic enzymes involved in biodeterioration, including cellulase, amylase, gelatinase, and pectinase. The conservation of the historical manuscript against fungal deterioration was achieved using innovative and pioneering environmentally friendly biomaterials: *Lactobacillus plantarum* extract (100 µg mL⁻¹) followed by green-synthesized TiO₂-NPs (100 µg mL⁻¹). Other conservation techniques, such as cleaning, consolidation, completion of missed parts, and other necessary methods for the studied manuscript, were also applied and revealed the aesthetic value of the manuscript.

**Supplementary Information:**

The online version contains supplementary material available at 10.1038/s41598-026-55481-1.

## Introduction

Historical manuscripts, books, and heritage artifacts are valuable national treasures found in archives, museums, and libraries around the world. Unfortunately, these materials are subjected to risk of deterioration due to microbial, chemical, and physical deterioration factors^1–3^.

Biodeterioration of manuscripts by fungi is one of the most important causes of the deterioration of library and archive collections, as it causes significant losses due to mechanical corrosion, enzymatic degradation, and acid corrosion, leading to distortion of aesthetic and structural value^4^. The colonization of fungi to papers in library and archives depending mainly on environmental factors and availability of nutrients especially carbon source^5^. The most common fungi that colonize papers belongs to xerophilic fungi which has the efficacy to grow in low water content. Papers enriched sources of carbon needed for growing fungi forming foxing or brown spots due to the secretion of saccharides, pigments, and acids which interact with paper contents^6,7^.

Traditional methods for managing microbial deterioration, especially fungal deterioration, which is more widespread, such as the use of chemicals, volatile oils, and ozone, are problematic. Therefore, researchers urgently need to develop new, fast-acting, safe, effective, economical, and environmentally friendly agents that also do not negatively impact workers, ink, or paper fibers^8^.

Probiotic strains are live microbes, nonpathogenic, and have health-promoting effects. These strains have the potential to produce a wide range of active metabolites that have antimicrobial features known as antimicrobial peptides, such as enzymes (lysozyme, phospholipase) and bacteriocins, and acids such as lactic acids^9^. Therefore, probiotic metabolites are considered a promising solution for controlling microbial deterioration due to their fast, safe, efficient, economical, and environmentally friendly nature. These strains typically come from genera like *Lactobacillus* and *Bifidobacterium*^10^. The most common probiotic strains is *Lactobacillus* ssp. that produce antimicrobial substances like lipopeptides, bacteriocins, and protease antibiotics^11^. Their ability to generate a wide range of metabolites makes them valuable for eco-friendly synthesis of stable nanomaterials.

Green synthesis of nanoparticles (NPs) using microbial strains depending on the quantity of metabolites in cell free filtrate which used as a reducing agent to transform metals to nanoscale structure^12,13^. These metabolites increase the stability of synthesized NPs and produce varied shapes and sizes to be integrated into wide applications. Due to the high probiotic metabolites, it is making these strains good candidates for green fabrication of NPs, like biosynthesized titanium dioxide nanoparticles (TiO_2_-NPs) with antifungal properties^14,15^.

Hence, the primary hypothesis of the present study is examining the effectiveness of the probiotic *L. plantarum* DSM-20174 strain extract and TiO₂-NPs synthesized by probiotic extract to biocontrol and conserve the biodeteriorated manuscript. To achieve this hypothesis, the historical manuscript was selected to assess the biodeterioration aspects using various methods, such as some analytical techniques (including SEM, ATR-FTIR, XRD, color changes, pH measurement, and photographic documentation), which were used for detection of the deterioration aspects before starting the conservation techniques. The historical manuscript underwent a series of conservation processes to ensure its preservation^16,17^.

This study aims to explain the deterioration mechanism of a historical manuscript dating back to 1158 AH, 1746 AD. It also aims to apply safe doses of eco-friendly substances (probiotic extract and biosynthesized TiO_2_-NPs) for controlling fungal strains that degrade a historical manuscript. This is the first research to explore this preservation strategy for historical manuscripts. Various conservation treatment methods are applied to protect manuscripts from deterioration while preserving the integrity of paper and leather materials.

## Materials and methods

### The historical background of the studied manuscript

The historical manuscript “Manh Elghfar Sharh Tnweer Elabsar” is a valuable document preserved at Al-Azhar Library in Cairo, Egypt. It dates back to 1158 AH, 1746 AD, and serves as a detailed reference in Islamic jurisprudence. The sample collection was achieved under formal permission from the Central Administration of Al-Azhar Library with code 93257 during Jun/2021. This manuscript was chosen for study due to its deteriorating condition. It was written in Arabic with iron gall ink, measures 25 × 18 × 11 cm, and consists of 680 paper sheets. The manuscript is identified by the documentation numbers 113506 (general) and 6214 (special). It was stored in closed iron cabinets without air conditioning, with a temperature of 27 °C and relative humidity over 60%, and poor ventilation in the storage area.

### Preparation of new Whatman Paper samples (control)

According to Abdel-Naser et al.^18^, a 24.0 cm diameter Whatman paper (No. 1) manufactured by the Whatman Company in England, with a cylindrical shape and an approximate radius of 12 cm, was utilized in this study. The new paper samples were employed to compare and analyze various properties of historical manuscripts to elucidate their deterioration mechanisms.

Whatman paper No. 1 was selected as the reference paper for the study due to its composition, containing a high percentage of pure cellulose (98% w/w) and the absence of additives, minimizing potential variables that could impact the experimental outcomes. With a thickness of 0.166 mm and an average diameter of 12.5 cm, this paper provides a stable and consistent baseline for comparison, making it a suitable choice for experimental studies in various fields.

### Vegetable-tanned leather samples (control)

The authors prepared new vegetable-tanned mimosa goatskin samples according to the procedure outlined by Abdel-Maksoud et al.^19^. These vegetable-tanned leather samples served as a control for comparison with the leather binding of the historical manuscript, aiming to highlight the distinctions in properties between the two samples produced under varying conditions. The samples were utilized as a control to investigate the degradation mechanism of the leather binding of the historical manuscript under study.

### Photographic documentation

The surface deterioration of the historical manuscript was examined through a combination of visual inspection and digital imaging. A Samsung 38 MP camera with an f/2.2 lens, manufactured in Japan, was utilized for this purpose. This comprehensive approach was crucial in identifying and describing the various aspects of deterioration present on the historical manuscript, which included both paper and leather binding. By employing both visual inspection and digital imaging techniques, a detailed analysis of the manuscript’s condition was achieved, providing valuable insights into its preservation and conservation needs^14^.

### Fungal strains associated with manuscript: isolation and identification

The heavily deteriorated areas (papers and leather binding) were touched with sterilized cotton swabs and used as a source for fungal isolation. Each swab was placed in a test tube containing 500 mg L^-1^ of sterile saline solution overnight to fungal spores are revived. After that, 100 µL of sterile saline solution was spread on the surface plates of Czapeck Yeast Extract (CYA) and/or potato dextrose agar supplemented with chloramphenicol (500 mg L^-1^ to inhibit bacterial growth. The inoculated plates were incubated for 8 days at 25 °C and observed daily to reinoculate the appearance of the fungal colony to a new plate for purification. The purified fungal strains were preserved on a slant for future study. The primary identification of obtained fungal isolates was achieved based on morphological, microscopic, and cultural identification using standard keys for *Aspergillus* spp.^20^, *Penicillium* spp.^21^, *Curvularia* spp.^22^, *Cladosporium* spp.^23^, and *Paecilomyces* spp.^24^.

To confirm the fungal characterization, ITS sequence analysis was used. The extraction of genomic fungal DNA was achieved based on Gene Let genomic DNA kit (Thermo) protocol. PCR was used to amplify the ITS region by ITS1 (5 `-TCCGTAGGTGAACCTGCGG-3 `) and ITS4 (5 `- TCCTCCGCTTATTGATATGC-3 `) primers ^25^. The mixture of PCR (50 µL) containing PCR-Master Mix (Thermo), primer (0.5 µM of each one), extracted fungal DNA (1µL). The PCR protocol was as follows: hot starting cycle for 3 min at 94 °C, followed by 30 cycles for 30 Sect.  (94 °C), 30 Sect.  (55 °C), 1 min (72 °C), and final cycle for 10 min at 72 °C. ABI-3730 × 1-DNA sequencer was used for sequencing analysis and compared the obtained sequences with those deposited on Gene Bank by NCBI-BLAST program. Finally, the phylogenetic tree was drawn by MEGA v6.1 software (www.megasoftware.net) bootstrap analysis. The species identification was based on combined morphological and ITS-based molecular evidence, not ITS similarity alone. We carefully re-evaluated all isolate assignments; where species-level certainty was less strongly supported by combined evidence, identification has been conservatively revised to genus level.

### Enzymes activity

The ability of various fungal strains isolated from deteriorated historical documents and leather bindings to secrete different enzymes, including cellulase, amylase, gelatinase, and pectinase, was investigated using the agar plate method. Each fungal strain was inoculated on mineral salt agar (MSA) media supplemented with specific substrates (CMC for cellulase, starch for amylase, gelatin for gelatinase, and pectin for pectinase) and incubated for 72 h at 25 °C ^26^. After the incubation period, each plate was flooded with Congo red for cellulase, iodine solution for amylase, HgCl_2_ solution for gelatinase and pectinase, and the results were recorded as the diameter of clear zones around fungal growth in millimeters.

### Biodeterioration assessment

#### Environmental scanning electron microscopy (ESEM)

The surface morphology of the control and historical paper and vegetable-tanned leather samples was examined using an environmental scanning electron microscope (ESEM)^27^. A Quanta 3D 200i from Boynton Beach, FL, USA, with an FEI-accelerated voltage of 20.00 kV was utilized for the analysis. The samples were observed under low vacuum conditions and without any prior preparation. This investigation took place at the Conservation Centre of the Grand Egyptian Museum in Giza, Egypt.

### Attenuated total reflection/fourier transform infrared spectroscopy (ATR/FTIR)

The Attenuated Total Reflection–Fourier Transform Infrared Spectroscopy (ATR-FTIR) analysis of both the new and historical paper and leather samples was carried out using a Bruker Vertex 70 instrument. The analysis covered a range of 4000–400 cm^− 1^ with a resolution of 4 at the Archaeological Research and Preservation Centre, Supreme Council of Antiquities, Ministry of Tourism and Antiquities.

### X-Ray diffraction analysis (XRD)

XRD analysis (PANalytical X’Pert Pro PW3040/60) was used to evaluate the cellulose crystallinity of historical paper in relation to the control (Whatman No. 1). Gonio was chosen as the scan axis. The generator specifications were 40 mA, 45 kV, and a 240 mm goniometer radius. Copper (Cu) was used as an anode material. The analysis was conducted in the Conservation Centre, Grand Egyptian Museum CC-GEM), Giza, Egypt^28^. The following formula was used to calculate the crystallinity index:

1 $${\mathrm{I}_{\mathrm{c}\mathrm{r}\mathrm{y}\mathrm{s}}} = \frac{{{\mathrm{I}_{002}} - {\mathrm{I}_{\mathrm{a}\mathrm{m}}}}}{{{\mathrm{I}_{002}}}} \times 100$$

Where I_Crys_ is the crystallization index, I_002_ is the intensity at a 2θ value of 22.6º, and I_am_ is the intensity at a 2θ value of 19º.

### Measurement of color change

The color change in all samples was assessed using the CIE*Lab system to determine the formula:

2 $$\Delta \mathrm{E} = \sqrt {{{(\Delta \mathrm{L})}^2} + (\Delta \mathrm{a}{)^2} + (\Delta \mathrm{b}{)^2}}$$

The L* scale measures lightness from 0 (black) to 100 (pure white). The a* parameter represents the position on the red-green axis (+ a for redder, -a for greener), while the b* parameter indicates the position on the yellow-blue axis (+ b for more yellow, -b for bluer). The total color difference (ΔE) between the two samples. The measurement was made using a portable spectrophotometer by Hunter Lab-Reston, VA, USA. The measurement was carried out according to El-Gamal et al. ^29^.

### Measurement of pH value

In accordance with Wouters et al., the pH value of the leather historical samples was calculated and compared to control samples^30^. But with some modifications. The pH levels of the leather samples were measured by immersing 1 g of leather in 50 ml of deionized water for about 6 h to enable the ions to dissolve into the liquid. The pH levels were gauged with a waterproof AD12 pH meter set between 2 and 7, at 21–22 °C.

According to Abdel-Nasser et al., pH values of the historical paper manuscript and the control (Whatman No. 1) were measured^31^. The surface pH was measured using a temperature meter with a flat electrode and an Adwa AD 1030 pH/mV. The pH readings were obtained by placing the flat electrode on paper without extracting a sample.

### Conservation study of the deteriorated manuscript using probiotic strain *Lactobacillus plantarum* and biosynthesized TiO_2_-NPs

The probiotic strain *Lactobacillus plantarum* DSM-20174 and biosynthesized TiO_2_-NPs (fabricated by this probiotic strain) were used to apply on deteriorated manuscript for control against fungal growth. This strain originated at the Microbiological Resources Centre (MICRECEN) of the Faculty of Agriculture at Ain Shams University, Cairo, Egypt. This strain has the ability to produce antimicrobial substances such as lipopeptides, bacteriocins, and protease antibiotics^11^. Their ability to produce bacteriocins further enhances their role by providing antimicrobial protection. Bacteriocins are substances that are effective against a broad range of spoilage microorganisms and pathogens^32^.

In this study, historical deteriorated manuscript was treated by crude extract of *L. plantarum* followed by biosynthesized TiO_2_-NPs as eco-friendly approach for conservation of deteriorated manuscript against fungal deterioration.

To achieve this goal, a single colony of *L. plantarum* was picked and inoculated into MRS broth media (Ready-prepared, Merck, Germany) before being incubated at 35 ± 2 °C for 24 h. After that, the inoculated media was centrifuged at 1000 *rpm* for 10 min to separate cells from supernatant.

The collected supernatant was subjected to rotary evaporation to concentrate and collect the residue which was used after that with a concentration of 100 µg mL^–1^(safe dose based on our previous study according to biocompatibility test toward human normal cell lines)^31^ for spray on deteriorated manuscript (paper and leather) to control the fungal growth.

For green synthesis of TiO_2_-NPs, the collect probiotic cells were rinsed twice with sterilized distilled water before resuspending approximately 10 g of cells into 100 mL of distilled water. The resulting mixture was incubated at 35 ± 2 °C for 24 h. At the end of the incubation period, the mixture was centrifuged to collect the cell-free filtrate, which was used for the biosynthesis of TiO_2_-NPs by mixing with Ti[OCH(CH_3_)_2_]_4_ (metal precursor) under stirring conditions for 1 h to get a final concentration of 5 mM. The pH of the mixture was adjusted to 8 using 1N NaOH added drop by drop. The color change from pale yellow to white precipitate indicated the successful formation of TiO_2_-NPs. After 24 h of incubation, the white precipitate was collected, washed thrice with high-purity water (Milli-Q), and subjected to oven-drying at 200 °C for 3 h^33^. After collect the TiO_2_-NPs powder, a concentration of 100 µg mL^–1^ dissolved in DMSO, (safe dose based on biocompatibility assay against human normal cell lines according to our previous published literature)^34^, was applied on deteriorated manuscript for investigate the conservation process.

Biological control was carried out in the conservation laboratory at Al-Azhar Library using the manual spray method.

### Statistical analysis

The means of three independent replicates are used to represent the data collected for this study, and the statistical program SPSS v17 is used to analyze the findings using ANOVA. The Tukey HSD test was used to compare the mean differences between the treatments at *p* < 0.05.

## Results and discussion

### Photographic documentation

**Fig. 1:**
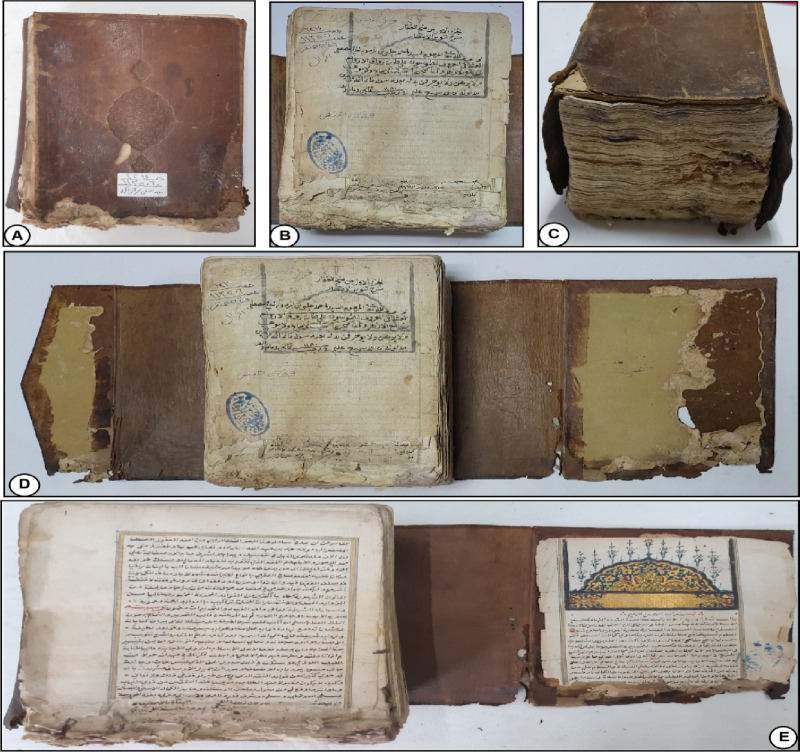
The historical manuscript studied at the Al-Azhar Library in Cairo, Egypt: (**A**,** C**, and** D**) The deterioration forms of the leather binding of the manuscript, (**B**,** D**, and** E**) The deterioration forms of the paper sheets of the manuscript.

The leather binding showed several forms of deterioration, as depicted in Fig. 1A, C, and D. Common signs included hardness, reduced flexibility, erosion of tanning material, loss of distinct sections, dust accumulation, missing parts, and stains from sources like fungi or pollution. Labels taped with pressure-sensitive tape were found to contribute significantly to the deterioration. Color variations and shrinkage were also observed.

The deterioration mentioned above is primarily due to external environmental conditions that are beyond control. Research has shown that vegetable-tanned leather is most susceptible to damage in an uncontrolled environment, which aligns with the overall findings. It is worth noting that the deterioration mechanisms of vegetable-tanned leathers involve hydrolytic and/or oxidative degradation^35^. Hydrolytic damage is caused by acidic air pollution containing SO_2_ and NO_2_, while oxidative damage is triggered by heat, light, and oxidative pollutants. Additionally, Carsote et al. demonstrated that the fibers of vegetable-tanned leather contract in response to fluctuations in temperature and relative humidity (RH)^35^. The historical manuscript papers exhibit various forms of deterioration, including writing distortions, mold stains, foxing, rust spots, dust, missing parts, library seals, and boreholes of different sizes. Additionally, there are tiny holes and discoloration present on the paper sheets (Fig. 1B, D, and E). The yellowing of the papers is an early sign of aging and degradation, which may progress to a brown color and brittleness over time, depending on the paper type and storage conditions^36,37^.

### Fungal isolation and identification

For historical manuscripts (papers and leather binding) that had degraded, the fungal community was isolated. Thirteen fungal isolates (designated AT.1–AT.13) were extracted from degraded samples (six fungal isolates associated with deteriorated papers and seven strains associated with leather binding). The fungal isolates obtained were examined using microscopic and cultural features for traditional identification (Table 1, Fig. S1, see supplementary data). The data showed that the isolates obtained belonged to the Ascomycota division. According to the conventional identification method, isolate AT.1 was identified as *Aspergillus chinensis*, while fungal isolates AT.8, AT.9, AT.11, AT.12, and AT.13 were identified as *A. flavus*. It was determined that fungal isolates AT.4, AT.6, and AT.7 were *Paecilomyces* sp. The *Penicillium* spp. isolated from deteriorated papers were identified as *Penicillium chrysogenum* for isolate AT.5. Additionally, fungal isolates AT.2 were identified as *Curvularia* sp., while isolates AT.3 were identified as *Curvularia tamilnaduensis*. The fungal isolates AT.10 were identified as *Cladosporium velox*.

**Fig. 2:**
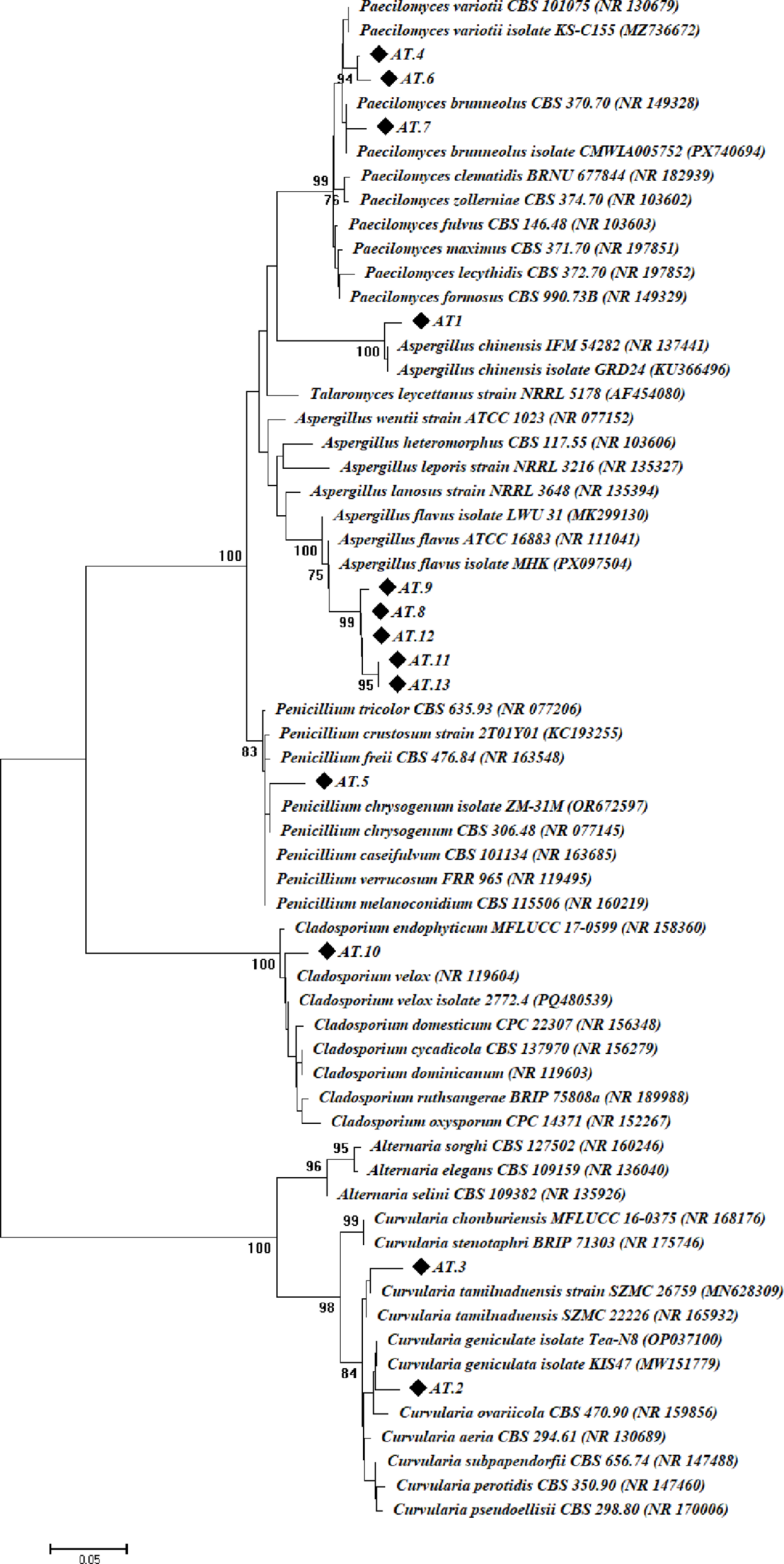
Phylogenetic tree of the obtained different fungal strains based on ITS sequence analysis against reference sequences on NCBI (National Center for Biotechnological Information). AT.1 – AT.13 refers to the ITS sequence of the isolated fungal strains from deteriorated historical manuscript in the current study. The tree was created by the neighbor-joining technique with a bootstrap value of 1000 replicates.

ITS sequence analysis was used to confirm the traditional identification of obtained fungal strains. Data reported in Table 1 and representative in Fig. 2 showed ITS sequence identification of the obtained fungal strains. The molecular analysis confirmed that the fungal strain AT.1 identified as *A. chinensis* with similar percentage of 99.01% with isolate has an accession number of NR137441. Fungal strains coded AT.2 and AT.3 were identified as *Curvularia geniculate* and *C. tamilnaduensis* respectively with similar percentages more than 98%. There are three strains of *Paecilomyces*, one obtained from deteriorated paper (AT.4) and identified as *P. variotii*, and two obtained from deteriorated leather (AT.6 and AT.7) and identified as *P. variotii* and *P. brunneolus*. On the other hand, *P. chrysogenum* (AT.5) and *C. velox* (AT.10), each one is represented by one strain with similar percentages of 98.46% and 98.64% with strains have accession numbers of NR077145 and NR119604 respectively. Molecular identification exhibited that the remaining five strains were identified as *A. flavus* with similar percentages more than 98% with strains deposited on Gene Bank (Table 1; Fig. 2). The obtained sequences in the current investigation were uploaded to Gene Bank with accession numbers of PZ255452 to PZ255464. Acknowledging the limitations of ITS for resolving some fungal genera and recommending future multilocus confirmation where necessary.

Based on the information gathered, *Aspergillus* spp. were the most prevalent fungi linked to the historical manuscript, accounting for 46.2% of all the strains that were obtained, followed by *Paecilomyces* spp. at 23%. Among *Aspergillus* spp., *A. flavus* accounted for 83%, followed by *A. chinensis* at 17%. *Curvularia* spp., *Paecilomyces* spp., and *Cladosporium velox* were the most prevalent fungal strains found in degraded old papers, based on conventional fungal identification, whereas *Aspergillus* species were the only fungal strains connected to leather binding (Table 1). In a similar study, twenty fungal isolates were isolated from deteriorated from deteriorated historical manuscript dated back to the 19th century and identified using morphological and cultural methods^38^. The author reported that the most prevalent fungal isolates were *Aspergillus* spp. with percentages of 45% (from the total), followed by *Penicillium* spp., *Eurotium* spp., and sterile hyphae (unable to form a distinguish structure) with percentages of 35%, 5%, and 15%, respectively. In another study, thirteen fungal strains were isolated from deteriorated parts of a historical manuscript dated back to 17th century and identified by cultural and microscopic examination to *Aspergillus niger* (3 isolates, 23%), *A. fumigatus* (2 isolates, 15.4%), *A. quadrilineatus* (3 isolates, 23%), *Penicillium citrinum* (2 isolates, 15.4%), and *P chrysogenum* (3 isolates, 23%) ^39^. Moreover, six fungal strains, *Fusarium poae*, *Wallemia sebi*, *A. tamarii*, *A. fumigatus*, *Cladosporium cladosporioides*, and *Eurotium chevalieri*, were isolated from historical leather binding dated back to 18th century^40^.

**Table 1:** Fungal community associated with the historical manuscript identified by traditional and molecular methods.

Fungal code	Source of isolation	Traditional identification	ITS identification	Closest accession number with similarity %	Gene bank accession number
AT.1	Paper	*Aspergillus chinensis*	*Aspergillus chinensis*	NR137441 (99.01%)	PZ255452
AT.2	Paper	*Curvularia* sp.	*Curvularia geniculata*	OM262223 (98.56%	PZ255453
AT.3	Paper	*Curvularia tamilnaduensis*	*Curvularia tamilnaduensis*	NR165932 (98.83%)	PZ255454
AT.4	Paper	*Paecilomyces* sp.	*Paecilomyces variotii*	NR130679 (98.8%)	PZ255455
AT.5	Paper	*Penicillium chrysogenum*	*Penicillium chrysogenum*	NR077145 (98.46%)	PZ255456
AT.6	Leather binding	*Paecilomyces* sp.	*Paecilomyces variotii*	NR130679 (98.2%)	PZ255457
AT.7	Leather binding	*Paecilomyces* sp.	*Paecilomyces brunneolus*	NR149328 (98.99%)	PZ255458
AT.8	Leather binding	*Aspergillus flavus*	*Aspergillus flavus*	NR111041 (98.8%)	PZ255459
AT.9	Leather binding	*Aspergillus flavus*	*Aspergillus flavus*	NR111041 (98.39%)	PZ255460
AT.10	Paper	*Cladosporium velox*	*Cladosporium velox*	NR119604 (98.64%)	PZ255461
AT.11	Leather binding	*Aspergillus flavus*	*Aspergillus flavus*	NR111041 (98.39%)	PZ255462
AT.12	Leather binding	*Aspergillus flavus*	*Aspergillus flavus*	NR111041 (98.8%)	PZ255463
AT.13	Leather binding	*Aspergillus flavus*	*Aspergillus flavus*	NR111041 (98.16%)	PZ255464

### Enzyme activity

Fungi are believed to be the primary degraders of historical manuscripts, due to their ability to live inside paper fibres and produce hydrolytic enzymes such as cellulase, amylase, xylanase, and pectinase, as well as necessary acids or pigments^40^. To reduce ink spread and improve fibre connectivity, the paper industry uses proteins, polysaccharides, gelatin, starch flour, and other synthetic materials in addition to organic materials (mainly cellulose). By releasing extracellular hydrolytic enzymes, fungi can decompose organic compounds and other additions, weakening and altering their structure^41^. The ability of culture-capable fungal strains to secrete a range of hydrolytic enzymes, such as cellulase, amylase, pectinase, and gelatinase, was examined in the current study. According to data analysis, all obtained fungal strains, except *Paecilomyces variotii* AT.4 (isolated from paper) and *P. variotii* AT.6 (isolated from leather binding), have the efficacy to release cellulase, amylase, and gelatinase to varied degrees. In contrast, all fungal strains that were isolated from leather binding and historical paper were able to secrete the enzyme pectinase (Table 2, Fig. S2 and S3, see supplementary data). According to data analysis, fungal strain *A. chinensis* AT.1 had the highest cellulase activity, with an inhibition zone of 50.5 ± 1.0 mm, followed by *C. tamilnaduensis* AT.3, *P brunneolus* AT.7, and *A. flavus* AT.8 with inhibition zones of 15.7 ± 2.3, 6.3 ± 0.6, and 5.7 ± 1.2 mm, respectively (Table 2, Fig. S2A). Intriguingly, fungal strains AT.7 and AT.10 differed significantly (*p* < 0.001) in cellulase activity, with inhibition zones ranging from 6.3 ± 0.6 mm to 8.0 ± 1.0 mm. In a recent study, *Aspergillus niger* and *Penicillium chrysogenium* were isolated from a historical manuscript from the 17th and 18th centuries, and they were chosen as the primary cellulase producers^42^. Once fungus colonies penetrate the paper fibers, hyphae result in physical alterations in addition to the buildup of fungal metabolic products in the fibers^43^.

Fungal strains *Paecilomyces brunneolus* AT.7 followed by *Penicillium chrysogenum* AT.5 are recorded as the highest amylase producer with clear zones of 14.0 ± 3.6 and 13.3 ± 1.5 mm, respectively. Whereas the lowest fungal amylase producer was recorded for *A. flavus* AT.11 and *A. chinensis* AT.1 with clear zones in the ranges of 3.0 to 4.0 mm (Table 2, Fig. S2B). In opposite, these strains (*A. chinensis* AT.1 and *A. flavus* AT.11) have the ability to secrete highest gelatinase enzymes with clear zones, measuring 28.0 ± 1.4 and 15.7 ± 2.1 mm, respectively, while AT.4 and AT.6 have no gelatinase activity (Table 2, Fig. S2C). Regarding pectinase activity, fungal isolates *Curvularia geniculate* AT.2 and *Penicillium chrysogenum* AT.5 were the highest producers, with clear zones of 17.7 ± 1.2 and 13.7 ± 1.5, respectively. Other strains were also pectinase producer with varied clear zones as shown in Table 2 and Fig. S2D.

To break down large molecules into smaller ones, hydrolytic enzymes such as cellulase and amylase convert cellulose and starch into glucose monomers. Pectinase and Gelatinase are enzymes that can break down a variety of proteins, including fibroin, collagen, and keratin, which are used to make silk, wool, and parchment, respectively^44^. The ability of fungal strains to produce a wide range of enzymes is necessary to understand their role in the biodeterioration of the text under study, as indicated by the results obtained. The manuscript (leather binding and paper) is associated with these strains. Previous studies have shown that the environmental parameters of the current study—60% relative humidity and a maximum temperature of 27 °C—facilitated the growth of fungi and created ideal conditions for the secretion of various active metabolites, including acids and enzymes^45^. The isolated fungal strains are unique in that they can secrete acidic metabolites in addition to producing a wide range of enzymes. The acidic secretions cause the acid hydrolysis of manuscripts. Borrego et al. demonstrated that fungal strains *Aspergillus terreus*,* A. niger*,* A. versicolor*,* A. ustus*,* Cladosporium sp.*,* Penicillium commune*,* P. chrysogenum*, and *P. citrinum* efficiently lower pH values by up to 4 through the release of acidic metabolites^46^. Due to these biodeteriorations, manuscripts lose their mechanical strength. Furthermore, the aesthetic alterations caused by pigment secretion may lead to information loss, material loss, and readability issues.

**Table 2:** Enzymatic activity of the obtained fungal strains isolated from deteriorated manuscript.

Fungal code	Enzymatic activity (mm)			
	Cellulase	Amylase	Gelatinase	Pectinase
*Aspergillus chinensis* AT.1	50.5 ± 1.0	4.0 ± 1.4	28.9 ± 1.4	15.0 ± 2.0
*Curvularia geniculate* AT.2	9 ± 1.0	4.3 ± 0.6	4.7 ± 1.1	17.7 ± 1.2
*Curvularia tamilnaduensis* AT.3	15.7 ± 2.3	9.3 ± 3.1	15.3 ± 0.6	6.0 ± 2.0
*Paecilomyces variotii* AT.4	0.0 ± 0.0	0.0 ± 0.0	0.0 ± 0.0	7.0 ± 1.0
*Penicillium chrysogenum* AT.5	19 ± 1.7	13.3 ± 1.5	7.7 ± 0.6	13.7 ± 1.5
*Paecilomyces variotii* AT.6	0.0 ± 0.0	0.0 ± 0.0	0.0 ± 0.0	5.7 ± 1.5
*Paecilomyces brunneolus* AT.7	6.3 ± 0.6	14.0 ± 3.6	7.3 ± 0.6	6.0 ± 3.6
*Aspergillus flavus* AT.8	5.7 ± 1.2	8.0 ± 1.0	8.7 ± 1.2	4.3 ± 2.1
*Aspergillus flavus* AT.9	5.7 ± 2.1	7.0 ± 2.0	8.7 ± 1.2	4.3 ± 1.5
*Cladosporium velox* AT.10	8.0 ± 1.0	6.0 ± 2.6	12.0 ± 1.0	8.7 ± 0.6
*Aspergillus flavus* AT.11	14.0 ± 1.7	3.3 ± 0.6	15.7 ± 2.1	8.3 ± 1.2
*Aspergillus flavus* AT.12	4.7 ± 2.1	6.3 ± 1.5	10.3 ± 0.6	8.7 ± 2.1
*Aspergillus flavus* AT.13	15.0 ± 1.0	5.3 ± 2.5	13.7 ± 2.1	13.3 ± 3.8

### Identification of ink used on the manuscript studied

#### Study of ink medium by ART-FTIR

A significant part of preserving the bond between ink and paper fibers involves studying the types of media that bind ink with manuscripts. To prevent the restoration process, cleaning, and treatment materials from affecting them and exposing them to decomposition and loss of writing inks for the manuscript, it is necessary to study and identify the types of inks. Appropriate restoration and treatment techniques are then chosen to treat and consolidate the inks.

In Fig. S4 (see supplementary data), the infrared spectrum analysis of gum arabic shows the basic functional groups involved in its formation. The (OH) bonds are observed at 3321 cm^–1^, the (C = C) bond appears at 1637 cm^–1^, and the (C-O) bond appears at 1040 cm^–1^. According to Apandi et al.^47^, it is revealed that gum arabic is used in the medium for black ink and red ink.

#### X-Ray diffraction analysis (XRD)

**Fig. 3:**
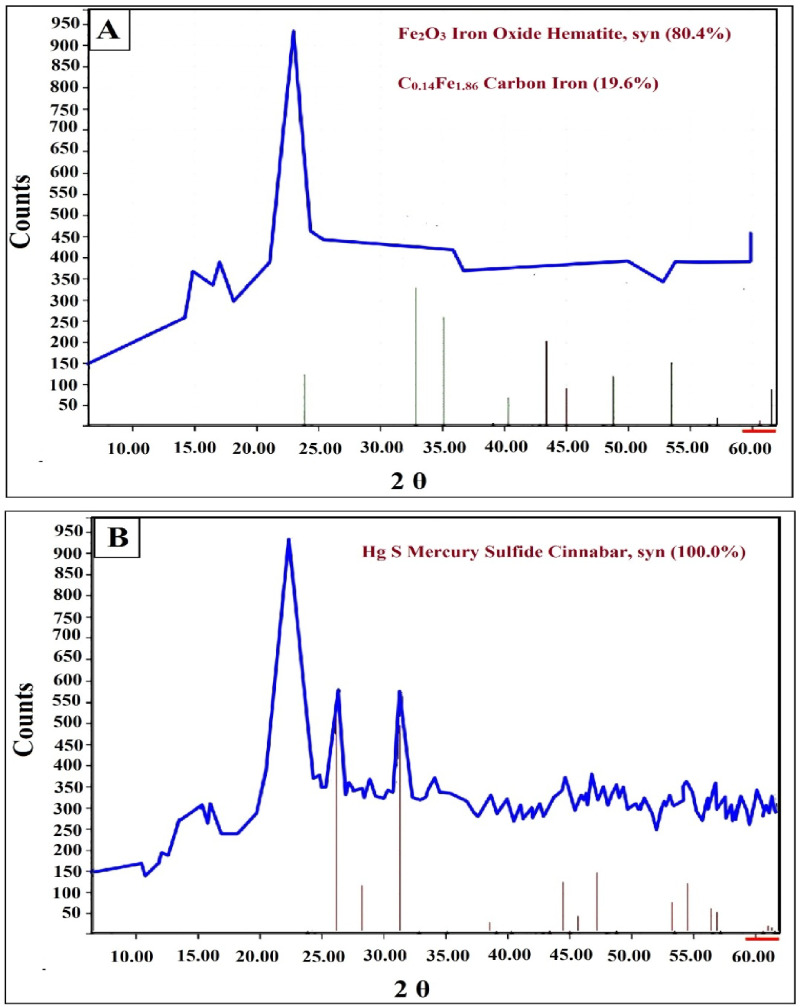
X-ray diffraction analysis of the inks used: (**A**) Black ink, (**B**) Red ink.

The X-ray diffraction analysis (Fig. 3) showed that the black ink was formed from iron carbon oxide (C.14 Fe1.86) by 19.6%, in addition to compounds of earth oxides such as hematite, which is iron oxide (Fe_2_O_3_) by 80.4% (Fig. 3A). These earth compounds are usually present in the inks as impurities due to the lack of sufficient purification of the ink. This type of ink was used in the eighth and ninth Hijri centuries of the Islamic era^48^. The red ink was made from a cinnabar compound, which is 100% mercury sulfide mineral (HgS) known as cinnabar (Fig. 3B). This mineral was used as a source for red ink by mixing it with pomegranate juice and adding a binder medium to it. The use of cinnabar was widespread in the Islamic era as a source for red ink^49^.

### Characterization of deteriorated aspects of the manuscript studied

#### Environmental scanning electron microscopy (ESEM)

**Fig. 4:**
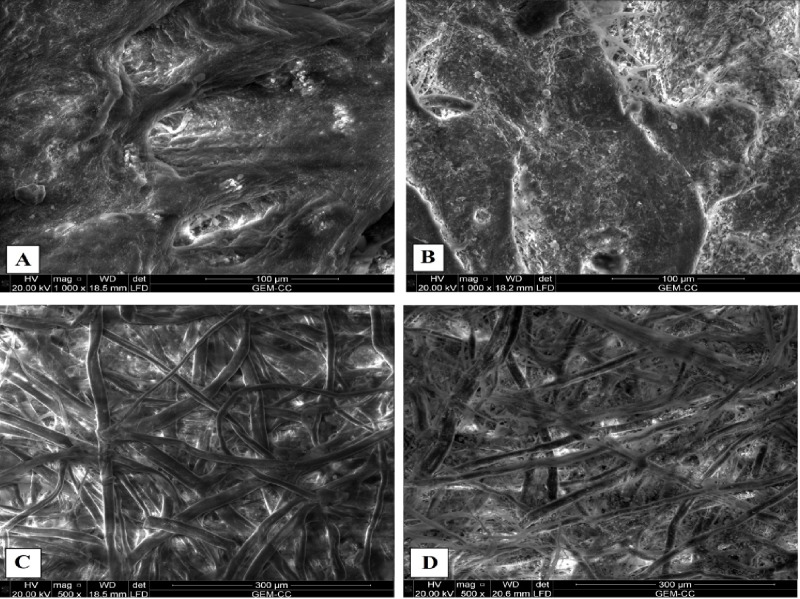
SEM images compare historical leather binding and papers to control samples: (**A**) leather sample (control), (**B**) historical leather, (**C**) Whatman paper (control), and (**D**) historical paper.

The new vegetable-tanned leather (control) (Fig. 4A) had a smooth surface morphology and a distinct clustering of coarse and fine follicles. In comparison to the control sample, the historical vegetable-tanned leather samples were primarily made from goat skin (Fig. 4B). The sample of historical leather had a rough, uneven surface, suggesting that the binding had deteriorated over time. This could be because the Al-Azhar library was in poor condition or because the examined manuscript was handled carelessly^50^. Historical leather surfaces may deteriorate as a result of dirt, folding, and other factors, in addition to the effects of soil during its deposition in the burial environment, handling during its use, and handling following excavation^51^. Additionally, they stated that it might not be possible to identify particular animal species based on the degree of surface deterioration and the details lost in the grain surface pattern. Moreover, the degradation of the surface in certain places may have resulted from the manuscript’s repeated improper handling. Additionally, there were some fine lines caused by insect invasion^51^.

These signs of deterioration can be linked to the activity of microorganisms, the state of the environment, or the treatment of the manuscript by workers or visitors, which results in the surface being destroyed in certain places^37^.

The current investigation also found that dust penetrated, accumulated, and adhered to the leather fibers, causing the grain surface pattern to vanish in a significant portion of the surface. According to Ebsen et al., filth, folding, handling during the leather’s life and after excavation, and soil contents during its deposition in the burial environment could all contribute to the deterioration of historical leather surfaces. Additionally, they stated that the loss of grain surface pattern characteristics can make it impossible to identify different animal species due to the extent of surface deterioration^51^.

In Fig. 4C, the Whatman fibers are shown as irregularly overlapping, complex, twisted ribbon-like structures with a slightly wrinkled surface. They have a broad, robust appearance, primarily associated with cotton fibers, as previously mentioned^52^. By comparing them to the control (Fig. 4D), it can be determined that the fibers of historical paper are identical to cotton fibers. Additionally, as previously reported, short rods (bacteria) or globe-shaped microbial growth (fungal growth) were found dispersed throughout the cotton fibers^53^. In some places, characteristics of the fibrous structure of cotton fibers preserved in old manuscripts have been lost, perhaps due to incorrect handling during the reading process. Additionally, it was observed that the fibers were weak, and each fiber’s width was smaller than that of the control fiber sample. Interestingly, the mycelium, hyphae, and spores of fungi were observed among the cotton fibers as short rods (bacteria) or globes (fungal growth)^53^. The environmental conditions, especially relative humidity, temperature, and pollution, encourage the growth of various fungal strains^54^. Additionally, each fiber’s width was found to be smaller than that of the control fiber sample. It was also observed that the fibers were weak. The paper’s surface also reveals significant damage to the paper, which could be the result of improper storage.

#### Attenuated total reflectance fourier transform infrared (ATR-FTIR)

**Fig. 5:**
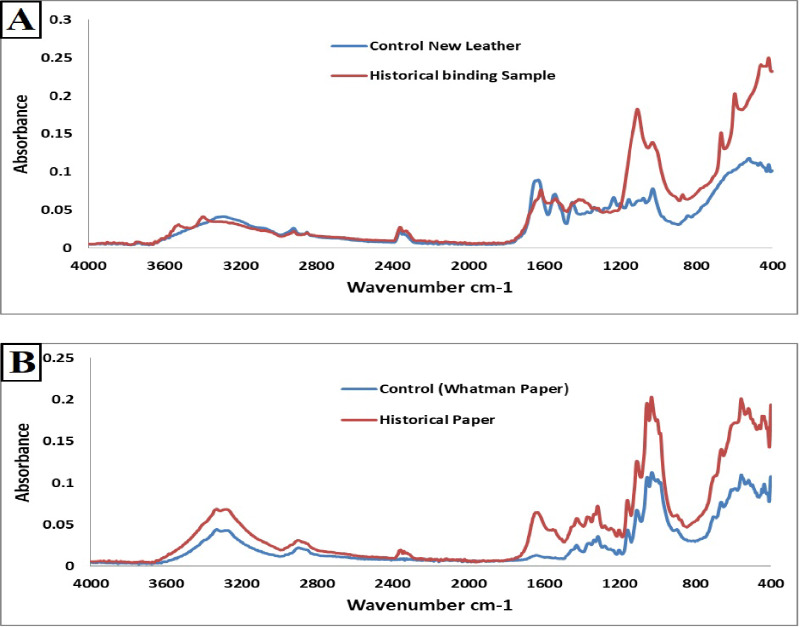
ART-FTIR analysis of new and historical leather samples: (**A**) The historical and new leather Samples, (**B**) The historical and Whatman paper samples.

According to the data, the collagen in the new and historical leather samples can be identified at wavenumbers 3294 cm^− 1^ and 2921 cm^− 1^ for the new sample, and at wavenumbers 3396 cm^− 1^ and 2919 cm^− 1^ for historical vegetable-tanned leather (Fig. 5A). The amide I band was detected at 1633 cm^− 1^ in the new vegetable-tanned leather sample and at 1619 cm^− 1^ in the historical leather sample. The stretching vibrations of the peptide carbonyl group (C = O), which are only weakly related to C-N stretching and N-H bending, are the main causes of the amide I band^55,56^. This is in line with Sebestyén’s assertion that the stretching vibrations of the peptide carbonyl group (C = O) combined weakly with C-N stretching and N-H bending are the main cause of amide I at the band indicated above^55^. It is sensitive to local order, and the protein molecule’s backbone conformation and hydrogen-bonding arrangement define exactly where it is located.

The N-H bending and C-N stretching vibrations were related to the amide II band at 1545 cm^− 1^ and 1542 cm^− 1^ for the new and historical vegetable-tanned leather samples, respectively. The bands of amide I and amide II are associated with the stretching of peptide N-H groups involved in inter-chain hydrogen bonding^57^.

According to Vichi et al., calcium carbonate was frequently discovered in historical leather as a result of the carbonation of calcium hydroxide leftovers from the liming bath with CO_2_ introduced during manufacturing or present in the atmosphere^58^.

The amide III bands at 1236 cm^− 1^ and 1273 cm^− 1^ for the new and historical samples, respectively, were linked to the proline side chain’s and glycine backbone’s N-H in-plane bending and CH_2_ wagging vibrations. The intensity of the amide III bands was roughly the same. The results showed that, in contrast to freshly vegetable-tanned leather, the collagen bands of older vegetable-tanned leather changed to increased and decreased value. This might be because the previously mentioned physical, chemical, and biological deterioration factors have caused the hydrolysis process to become oxidized.

However, notable differences were observed between the historical sample and the Whatman paper (control sample) (Fig. 5B). The cellulose paper was detected at wavenumbers between 3000 and 3700 cm^− 1^. In both the control and historical paper samples, different sections of the cellulose structure associated with O-H vibrations were identified at wavenumbers of 3332.39 and 3290.69 cm^− 1^, and 3334.30 and 3290.24 cm^− 1^, respectively. The bands of the historical sample showed an increase in both wavenumbers and absorbance intensity. This band indicates the presence of hydroxyl groups and is assigned to the stretching OH vibrations. These findings provide evidence for both intra- and intermolecular hydrogen bonding^59^.

The wavenumber corresponds to C-H stretching vibrations in the 2700–3000 cm^− 1^ range. The absorbance intensity of the historical paper’s C-H band at 2905.45 cm^− 1^ was higher compared to 2899.88 cm^− 1^ for the new sample. The stretching vibrations of the methyl and ethyl groups v(CH_3_) and v(CH_2_) that concede as cellulose molecules are represented by these bands^60^.

The oxidation process in the historical sample may have caused the band at approximately 1701 cm^− 1^ to indicate the stretching vibration of the C = O carbonyl group. The bands at 1644.62 and 1635.03 cm^− 1^ for the new and historical samples referenced the O-H in-plane bending vibration and the H-O-H deformation vibration of adsorbed water, respectively, refer to a physically absorbed water molecule. Cocca and coauthors reported that the strong affinity of paper for water makes it difficult to perform an FTIR study on paper samples^61^. Furthermore, they observed that the absorbed and bound water band is situated in the carbonyl group region (between 1635 and 1670 cm^− 1^), and that it might occasionally be rather broad and obscure the carbonyl group bands. The bands of the control sample were wider than those of the new and historical samples, suggesting more water loss. The bands at wavenumbers between 1248 and 1580 cm^− 1^ identified the C-O-H and CH_2_ bending vibrations. The absorbance bands at these wavenumbers were higher in the historical sample compared to the new paper sample. Furthermore, distinct C-O stretching, C-O-C, and C-C-O bending vibrations were represented by the bands at wavenumbers between 900 and 1160 cm^− 1^. The historical sample showed an increase in wavenumbers and absorbance intensities at these bands compared to the new sample.

Cellulose’s structure is linked to its functional groups. The in-plane bending vibrations of H-C-H and O-C-H were referenced in the band at 1427 cm^− 1^. The C-H deformation vibration was identified by the band at 1360 and 1369 cm^− 1^ in both the new and old samples. For both new and old samples, the band at 898 and 896 cm^− 1^ represents the C-O-C, C-C-O, and C-C-H deformation modes and stretching vibration, respectively^62^. Approximately at 661 cm^− 1^, the band was used as a reference for the C-OH of the plane bending mode. The bands of absorbance intensities at the aforementioned wavenumbers were higher than in the new sample. It can be claimed that the historical samples increased band intensities showed signs of cellulose oxidation and hydrolysis activities. The effects of the physical and chemical elements indicated above on the paper under study may cause oxidation or hydrolysis processes. Munajad et al. provided support for these findings by showing that cellulose fibres can lose their adsorbed water through oxidation as a result of heat ageing, and that the degradation process accelerated over time^63^. Furthermore, the hydrolysis and oxidation of the cellulose in paper are indicated by the band with a wavenumber between 1400 and 1800 cm^− 1^^64^. Using the aforementioned information, it was observed that the bands of the historical paper sample shifted to lower wavenumbers and produced larger intensities than the Whatman paper sample. It was clear from this that the historical paper sample had been subjected to deterioration, which could have been brought on by physical, chemical, or biological processes.

#### X-ray diffraction analysis (XRD) for measurement of cellulose crystallinity

**Fig. 6:**
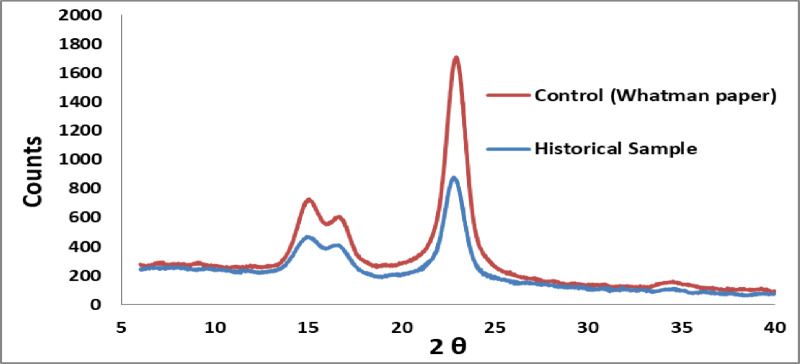
Crystallinity measurement of both new and historical papers using X-ray diffraction analysis.

Native cellulose is known to contain certain crystalline elements. Both the crystalline and amorphous portions of the fiber cell wall contain fibrils of cellulose molecules. According to Fig. 6, the Whatman paper sample had a crystallinity of 81.68%, whereas the historical paper sample had a crystallinity of 47.54%. Whatman paper samples usually have a high level of crystallinity because they contain more than 95% cellulose^65^. The findings demonstrated that the historical paper’s crystalline was noticeably less than the controls. This decline may result from a number of deterioration factors, including rising temperatures and falling moisture content, which damage and destroy the cellulose’s amorphous regions. Moreover, microorganisms aid in the formation of fossils and caked-on papers^66,67^. The results were consistent with claims that cellulose crystallization was inhibited in dry environments or when water was removed from cellulose materials^68^. According to Sandy et al., cellulose’s crystallinity can decrease as a result of increasing moisture and acid hydrolysis. However, cellulose crystallinity can also increase^65^. This is consistent with what Ioelovich stated that cellulose crystallization was inhibited in dry environments or when water was removed from cellulose materials^68^.

The historical paper’s crystallinity in the current study was far lower than the control’s. This decrease may be the result of a number of degrading processes, including the continuous rise in temperature and the drop in moisture content, which harm and disintegrate the cellulose’s amorphous regions. Additionally, microbes contribute to the creation of caked-on papers and fossils^67,69^.

#### Measurement of color change

**Fig. 7:**
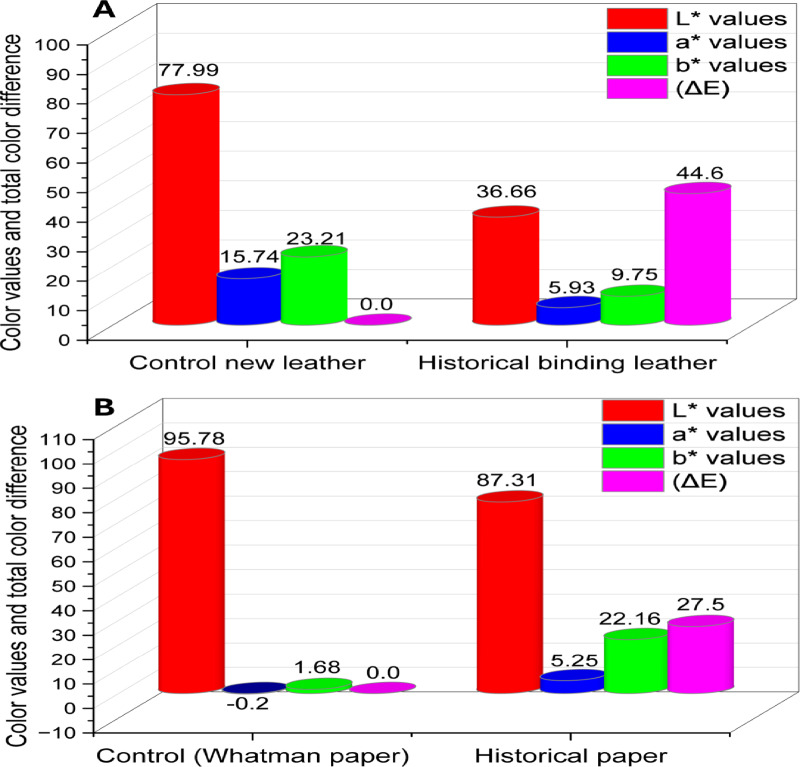
Color change measurement: (**A**) Control and historical leather, and (**B**) Control (Whatman paper No. 1) and historical paper.

The lightness (L*) of the new vegetable-tanned leather (Fig. 7A) was 77.99, while the historical leather binding was 36.66. The reduction percentage in the lightness of the historical sample was 53%. Both the new and historical leather tended to be in a red color. According to the data acquired, the historical sample’s a* value rose by 62%. The b* value was more yellow, according to the results. The b* value of the historical sample increased by 85% compared to the new vegetable-tanned leather. The overall color difference (∆E) for the historical sample was 44.6. The primary factors causing the change in color values (L, a, and b values) and the total color difference (ΔE) are variations in temperature and relative humidity, excessive light, pollution, and biological attack^70^.

The lightness (L* value) of the historical paper sample dropped to 78.31 from 95.78 for the Whatman paper control sample (Fig. 7B). In the historical sample, the L* value decreased by 18%. The historical sample’s a* value increased more than that of the control sample, growing by 96%. The control sample had a b* value of 1.68, while the historical sample had a value of 22.16. A comparison with the control Whatman paper revealed that the historical paper’s total color difference (ΔE) was 27.5, which was excessive. The creation of organic acids and pigments by fungi may be the cause of the color shifts. Moreover, microbial invasion results in white coatings and multicolored patches^71^. The aging process causes a hue to darken, and this darkening worsens over time^72^.

#### Measurement of pH value

The new vegetable-tanned leather sample had a pH of 4.1, while the historical leather binding sample had a pH of 3.2 (Fig. S5, see supplementary data). It was clear that the historical leather’s pH level had dropped considerably. Environmental contamination, excessive acid addition during tanning, or the enzymes or acids produced by bacteria or fungi can all cause the pH value of the historical sample to decrease.

The pH value of the historical paper, however, decreased from 7.5 for the control (Whatman paper) to 5.5 for the historical paper, suggesting that the historical manuscript has several issues associated with deterioration.

### Conservation study

#### Sterilizing of deteriorated historical manuscript

The papers were individually sprayed using *L. plantarum* extract at a concentration of 100 µg mL^–1^, followed by biosynthesized TiO_2_-NPs at a concentration of 100 µg mL^–1^ by placing the historical paper on a sheet of polyester fabric and sterilizing it using a hand sprayer. The historical paper was covered with another polyester fabric sheet, and the three layers of the upper polyester fabric sheet, historical paper, and lower polyester fabric sheet were then turned upside down. The other side of the historical paper was also sterilized using the same method. The sterilized historical paper was dried between the polyester and cardboard sheets under light pressure to prevent changes in dimensions during drying. Paper must be deacidified because acidity can harm it in various ways. For instance, substrates with acidity between 4.0 and 6.0 encourage the growth of fungi^73^(Fig. S6, see supplementary data). This aligns with the use of certain materials with antibacterial and antifungal properties, such as nanoparticles, to prevent this environmental degradation^74–76^. The probiotic bacterial strain, *L. plantarum*, is famous for active metabolites that have antimicrobial features. For instances, active metabolites, such as bacteriocin, lipopeptides, and protease enzyme, are produced by this strain^32^. Moreover, green synthesized TiO_2_-NPs are characterized by wide range of applications involved antimicrobial activity against different microbial strains^14^.

#### The leather binding conservation

On the leather, there were stains, dust, paper stickers, and polyethylene tape. In order to remove these stains, mechanical and chemical cleaning methods will be described in more detail below:

#### Dry cleaning of leather binding

On the inside of the leather, the adhesive tape that served as a substitute spine for leather bookbinding was carefully removed. To avoid the leather surface from flaking, the tape was completely removed from the outside face of the leather using a wet removal technique. Additionally, cardboard support was manually added in place of the leather support. Using a scalpel and a brush, the adhesive remnants of the historical leather support, as well as dirt, dust, and bug residues, were removed from both sides of the leather^77^.

#### Chemical cleaning of leather binding

We used an acetone solution to remove any leftover adhesive tape and residues from the leather surface. The paper tickets that had been stuck to the leather surface were taken off using a 1:1 ethanol and water solution. The method worked well and made removing the tickets easy. The stains on the leather surface could be effectively removed using wet cotton soaked in a mixture of distilled water and ethanol (70:30 v/v).

After cleaning, the leather was gently compressed between two pieces of cardboard to eliminate any remaining cleaning solution residue. This process is essential as it prepares the leather for absorbing softening chemicals. To counteract the surface cracking caused by the different expansion and shrinkage of the two leather surfaces during the evaporation of the cleaning solutions, it is important to apply pressure to the leather during drying and prevent it from arching or bending^78–80^.

#### Softening the leather bookbinding

After applying the mixture of ethanol, castor oil, and tea tree oil to the leather, another mixture had to be applied depending on the condition of the leather. In order to promote flexibility, softness, and prevent corrosion of leather edges, the second mixture, which contained lanolin, castor oil, and tea tree oil, was applied to the leather^81^.

To prevent folded edges and shrinkage areas in the leather, it was placed under the piston for 24 h between two sheets of polyester and two layers of cardboard. However, during the pressure, the layers of cardboard absorbed the overly flexible leather components. The leather is now prepared for the restoration phase at the conclusion of this step.

#### Restoration of the leather binding

For the restoration of the leather bookbinding’s spine, a strip of freshly vegetable-tanned goat skin with dimensions of 21 × 2.3 cm was created. To make the original edges wider, the new leather strip’s width was increased by approximately 0.5 cm. By measuring the width of the space between the flap and the lower board of a leather bookbinding and extending it by 6 mm, the size of the missing spine was estimated. The edges of the new leather strips were scraped to match the width of the parts applied to the old leather using a sharp knife^50^.

The inside edge of the top and lower board and the outer margins of the leather were painted with a 5% aqueous colloidal hydroxypropyl cellulose (Klucel G), ensuring not to use more adhesive than necessary. The two sides of the leather bookbinding were covered with two translucent polyester textiles and two pieces of cardboard, respectively. All layers were then compressed under the piston for around 24 h until the adhesive material is completely dry. During the drying process, the piston was used to prevent the leather from twisting and warping.

**Fig. 8:**
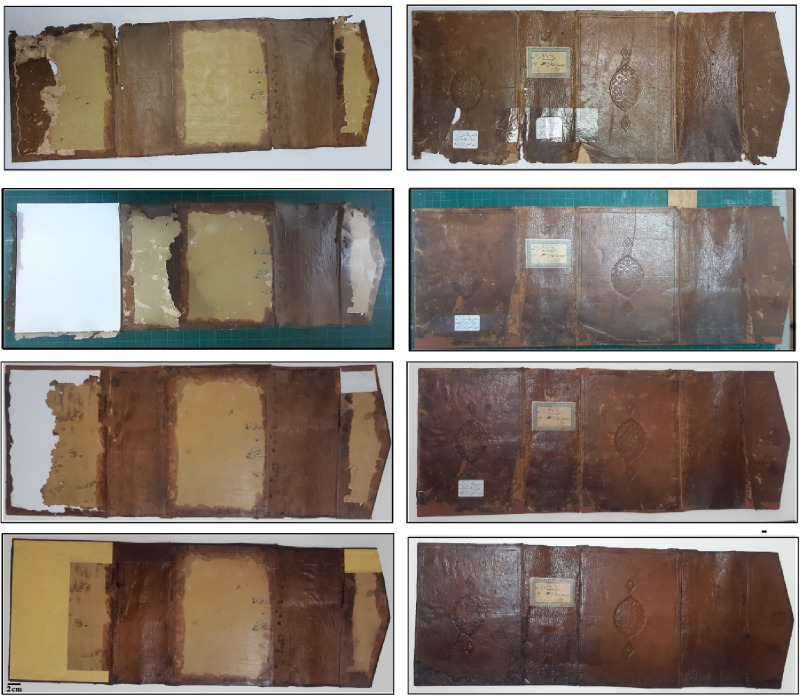
The leather binding after conservation.

Two pieces of acid-free cardboard were attached to the upper and lower boards of the leather bookbinding using hydroxypropylcellulose (Klucel G). After that, the fresh leather edges were folded and adhered to the inner face of the cardboard. The acid-free paper lining was applied to the inner face of the leather bookbinding using Klucel G and pressed under the piston until dry, as shown in (Fig. 8).

#### Paper conservation

##### Paper cleaning

The cleaning techniques used in this investigation were chosen based on the condition of the paper. Mechanical cleaning to remove the foreign materials was carried out before chemical cleaning, to avoid transferring the dust into the paper-matrix via cleaning solutions. Non-adherent dust and insect residue were removed from the delicate paper by gently brushing it in one direction. The dry mold conidia were mechanically removed using a soft brush and dry cotton swabs. Sediment, insect excrement, and other noticeable things that were present on the surface of some papers were removed using forceps and a needle^82^.

The paper spine dry adhesives, which caused the cut edges of the dual papers to solidify and become thicker than the other edges, were removed with a scalpel. Dry scrubbing with a sponge eraser removed the adherent dust and soot streaks. The area between the thumb and forefinger was gently wiped on the worktable’s smooth, dry surface while the part being cleaned was held between them. To control the paper on the work surface, the hand was not raised in the air. It was discovered that mechanical cleaning was a useful technique for getting rid of silt and other noticeable elements from the paper surface. The sponge eraser removed the soot stain, surface filth, and grime with great success. Moreover, it did not affect badly on the paper surface.

Wet and chemical cleaning is a desirable and suitable method for removing spots penetrating the fibers. Each area on the paper had its morphological shape visually assessed in order to identify its type and apply the appropriate remedy. To completely remove fungus spots and serve as a disinfectant, a cotton swab was moistened with a solution of ethanol: deionized water (70:30 v/v) ^80^. To ensure the ethanol’s antibacterial activity, the ethanol solution was in contact with the diseased surface for more than three minutes^83^. Acetone: ethanol (1:1 v/v) was used to clean the title page, and this solution produced good results for the cleaning of the paper as a whole. To prevent the paper dimensions from altering, the treated papers were dried under low pressure. Iron-gall ink is found to strongly penetrate paper fibres by Craddock, ^84^, however colored inks have little resistance to cleaning agents. As a result, the historical papers’ red ink spots weren’t cleaned with cleaning agents.

The manuscripts were shown to have stains from mildew, soot, dirt, and dust. The stains were removed using mechanical and chemical cleaning methods. Some of the tools and materials used in the cleaning procedures included soft brushes, water, and ethyl alcohol.

##### Paper restoration and consolidation

Indian-origin methyl cellulose and hydroxypropyl cellulose (Klucel G), both cellulose derivatives, were selected as the adhesive and consolidating materials, respectively. These materials were preferred due to their chemical similarity to cellulose^85^.

**Fig. 9:**
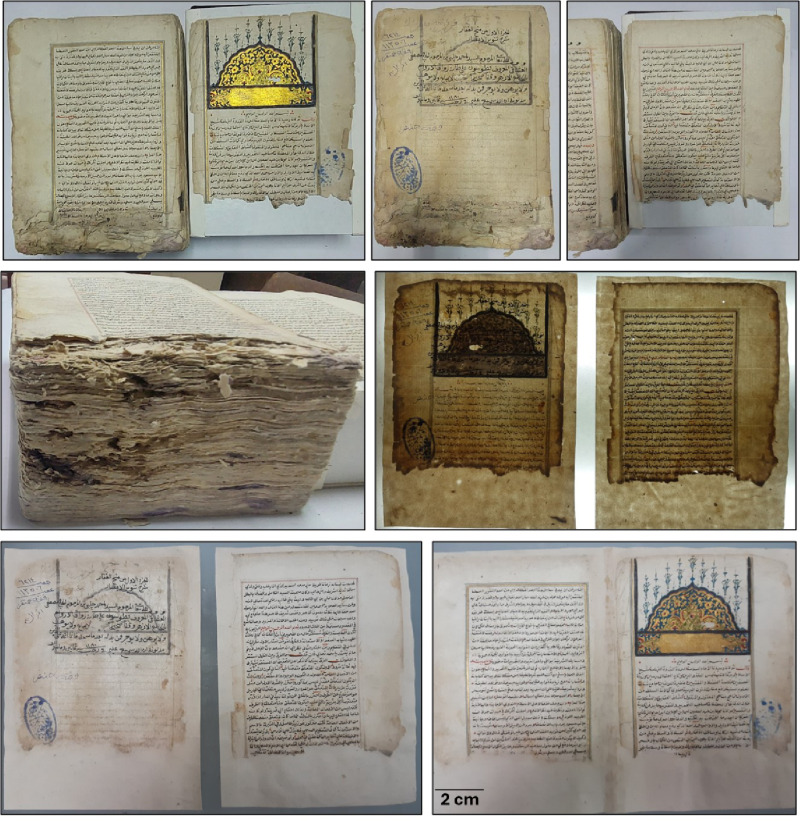
The restoration and consolidation of the historical paper manuscript.

Additionally, methyl cellulose is known for its resistance to chemical and biological deterioration^86^. To address missing pieces, fill large gaps, repair minor holes, and mend tears and cuts, paper pulp, Japanese paper, restoration paper (produced with a leaf casting machine), and methyl cellulose (3–4%) were used. These procedures were carried out using brush No. 1, piston, forceps, scalpel, spatula, and ruler. The strength of weaker paper was enhanced with Klucel G. The consolidating material was applied with a brush, as shown in Fig. 9.

The paper restoration and consolidation were carried out manually, taking into account the thickness of the historical paper, the characteristics of the adhesives, and the compatibility of the filling materials with the historical paper. The tears, cuts, and breaks have sharp edges and do not have free cellulose fibers. According to Jacob et al., a small brush and a slight adjustment were used to apply 3% MC to the transparent Japanese paper strip^87^. The strip was then placed on the cut and pressed until well adhered. Depending on the tear’s location, the widths of the strips for the text and margins ranged from 5 to 10 mm.

The folds of the dual paper were brought back together using a strip of Japanese tissue after the cuts of the paper spine had been cleaned and scraped with a knife to a width of 4 mm of the adhesion surface area^6^. When the historical and Japanese papers were pasted together using MC and pressed under a piston until dry, the fibers of the Japanese tissue were parallel to the fibers of the historical paper^86^. It was discovered that by carefully roughening the adhesive surface area with a scalpel, the adhesion grew stronger. It was discovered that by carefully roughening the adhesive surface area with a scalpel, the adhesion grew stronger.

Hydroxypropyl cellulose (Klucel G) was used to strengthen damaged paper. Two concentrations of hydroxypropyl cellulose (1.0% and 0.5% in water) were used for consolidating less degraded and more deteriorated paper, respectively^88^.

**Fig. 10:**
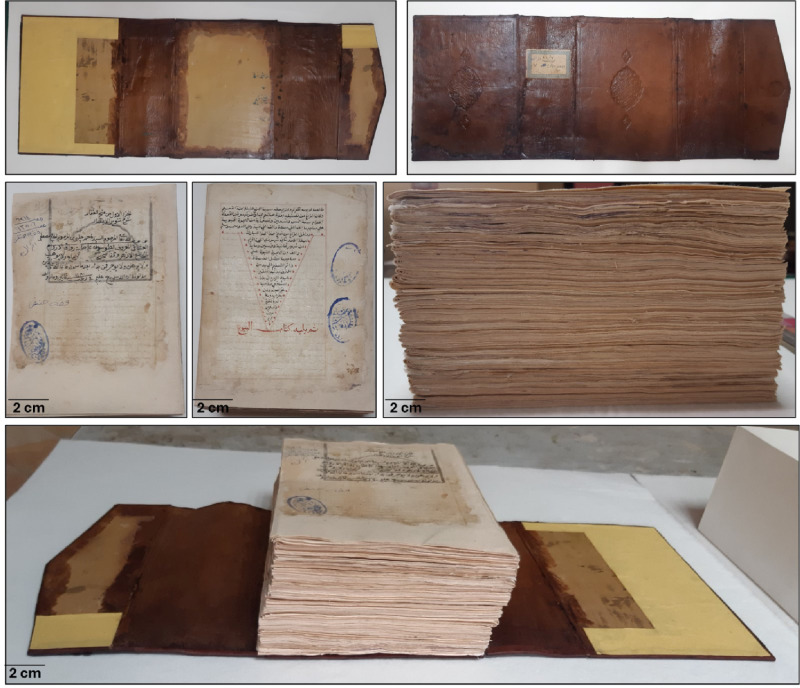
Historical manuscript after the conservation processes.

The layers were brushed and dried under pressure with the historical paper sandwiched between two layers of spun polyester. When Klucel G replaced the surface sizing layer that the weak paper had lost and enhanced the adhesive strength between fibers, the consolidated paper showed significant improvement in coherence (Fig. 10).

#### Preservation of the studied manuscript

In order to preserve the manuscript and protect it from various harmful factors of deterioration during storage, a preservation box made of acid-free cardboard was prepared. Most manuscripts are exposed to damage during preservation and storage in their storage places^89^. The cardboard used to make the folder was increased in size by 1 cm² on all sides so that the manuscript could be placed inside it securely. This was done after calculating the measurements of the manuscript in its final form following the various restoration procedures.

**Fig. 11:**
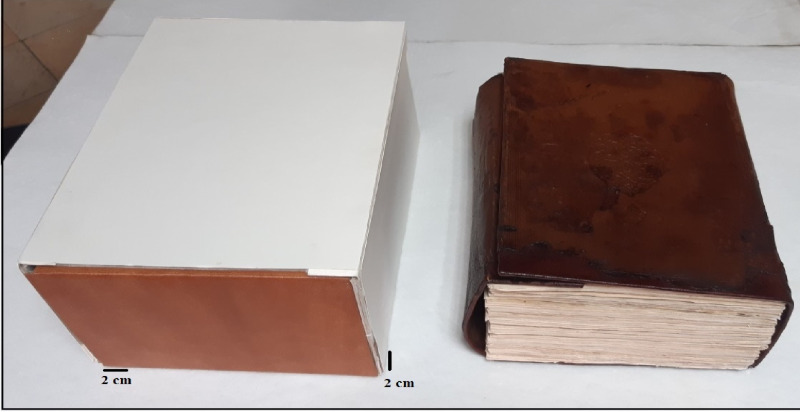
The historical paper manuscript and leather binding in final form after the conservation processes.

The components of the clipboard were adhered and cohered together using 5% hydroxypropyl cellulose glue. The method of preparing the clipboard and storing the manuscript is clarified, and this is evident through (Fig. 11).

## Conclusion

The historical manuscript studied showed significant degradation due to biodeterioration and improper storage conditions. Thirteen fungal strains linked to the manuscript’s deterioration have been identified. These fungi can secrete hydrolytic enzymes like cellulase, amylase, gelatinase, and pectinase, which play a role in biodeterioration. Most of the fungal strains can produce all necessary hydrolytic enzymes. Common signs of deteriorating leather binding include hardness, loss of flexibility, erosion of tanning material, dust accumulation, missing parts, and stains from pollution or fungal contamination. Comparison with control samples using photographic documentation, SEM, ATR-FTIR, XRD, pH values, and color measurement revealed significant deterioration in the historical samples. ATR-FTIR and XRD help identify the ink and medium used. The manuscript was sterilized with *Lactobacillus plantarum* DSM-20174 (100 µg mL^–1^) and its biosynthesized TiO_2_-NPs (100 µg mL^–1^) for preservation. Mechanical and chemical cleaning techniques using cotton, soft brushes, water, and ethyl alcohol were effective in preserving the manuscript. Tea tree oil in ethanol treated the leather binding, and ethyl alcohol mixed with calcium hydroxide nanoparticles removed paper acidity. Hydroxypropyl cellulose 3% (Klucel G) was used as an adhesive for manuscript restoration.

## Supplementary Information

Below is the link to the electronic supplementary material.

Supplementary Material 1.

## Data Availability

The datasets used and/or analyzed during the current study are available from the corresponding author upon reasonable request.
