# Supplementary material for: An integration of bacterial probiotic extract and green nanoparticles as a safe approach for conservation of historical manuscript against fungal deterioration: an applied study
Source: Sci Rep. 2026 Jun 6;16:17533. doi: 10.1038/s41598-026-55481-1 (PMC13242506; doi:10.1038/s41598-026-55481-1)
Supplement: Supplementary file 1 — Supplementary Material 1. [file 41598_2026_55481_MOESM1_ESM.docx]

**An Integration** **of Bacterial Probiotic Extract and Green Nanoparticles as A Safe Approach for Conservation of Historical Manuscript Against Fungal Deterioration: An Applied Study**

**Mahmoud Abdel-Nasser^1^, Amr Fouda^2^*, Gomaa Abdel-Maksoud^3, 4*^**

1. Department of Manuscripts Conservation, Al-Azhar Al-Sharif Library, Cairo 11511, Egypt.
2. Department of Botany and Microbiology, Faculty of Science, Al-Azhar University, Nasr City, Cairo 11884, Egypt.
3. Heritage Science Program, School of Humanities, Faculty of International Business and Humanities, Egypt-Japan University of Science and Technology (E-JUST), New Borg El-Arab City, 21934, Alexandria, Egypt.
4. Organic Materials Conservation Department, Faculty of Archaeology, Cairo University, Giza 12613, Egypt.

**Corresponding author:**

Amr Fouda ([amr_fh83@azhar.edu.eg](mailto:amr_fh83@azhar.edu.eg))

Gomaa Abdel-Maksoud ([gomaa2014@cu.edu.eg](mailto:gomaa2014@cu.edu.eg); [gomaa.abdel-maksoud@ejust.edu.eg](mailto:gomaa.abdel-maksoud@ejust.edu.eg)).

*
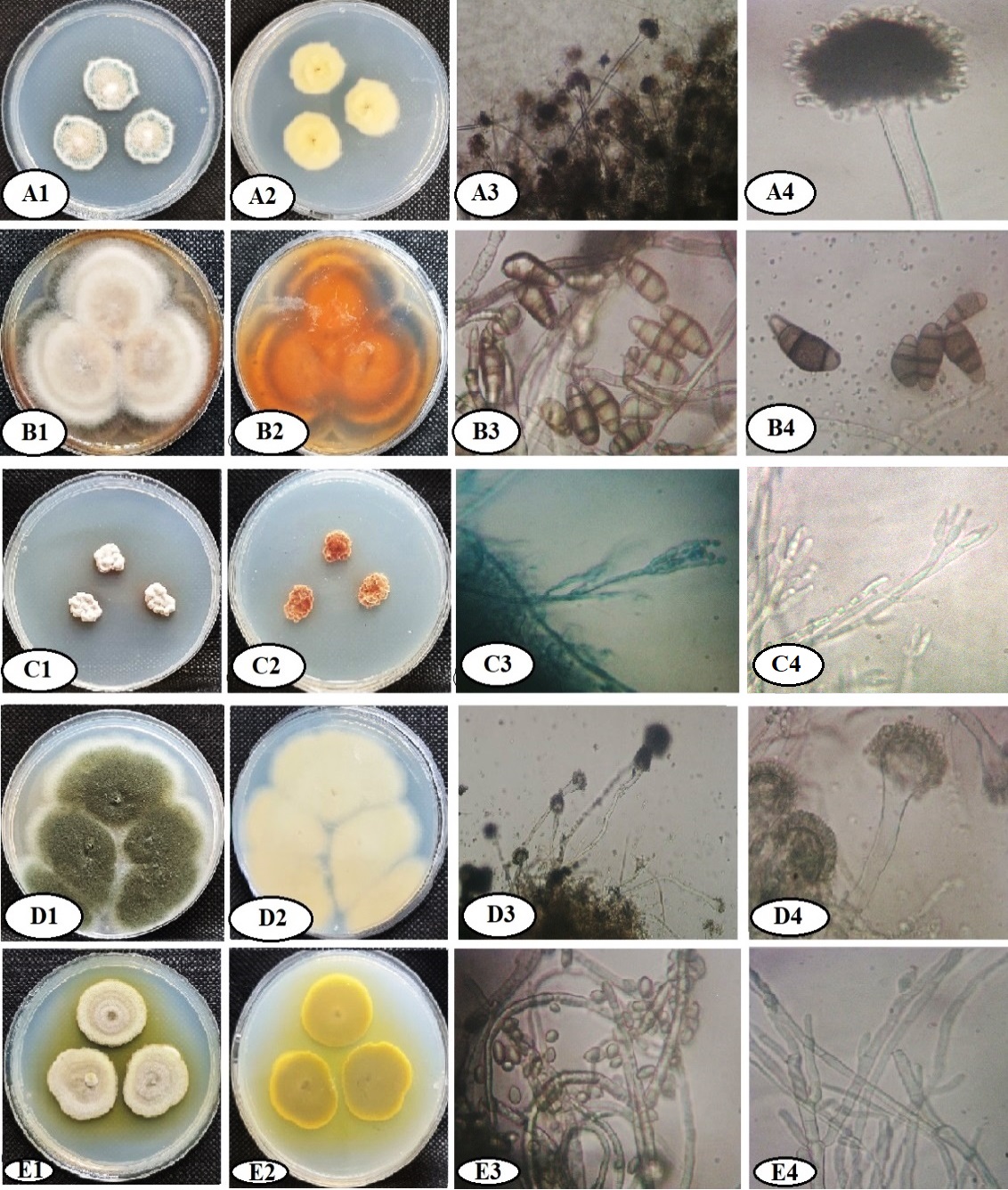
*

**Fig. S1.** Examination of the morphology traits of various fungal strains growing on the historical manuscript studied (papers and leather binding): (A) *Aspergillus chinensis*; (B) *Curvularia sp.*; (C) *Paecilomyces sp.*; (D) *Aspergillus flavus*; and (E) *Cladosporium velox*. (E1) represents a fungal colony; (E2) represents a reverse colony; (E3 and 4) show bright field microscopes (X = 800).


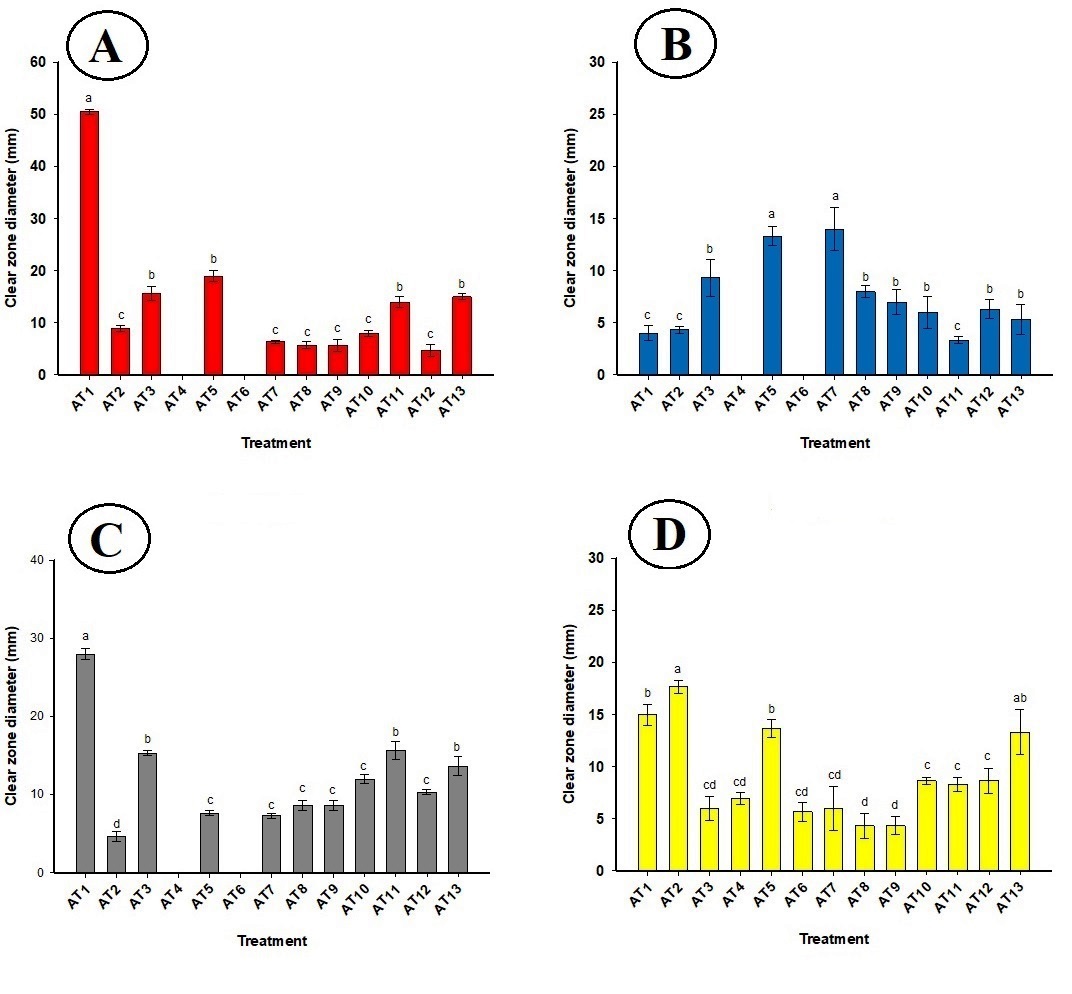
**Fig. S2.** Enzymatic activity of fungal strains isolated from the historical manuscript: (A) Cellulase enzyme, (B) Amylase enzyme, (C) Gelatinase enzyme, and (D) Pectinase enzyme. Different letters between various fungal strains on bars for the same enzyme indicate that the mean values of the clear zone are significantly different (p ≤ 0.05) (n = 3).


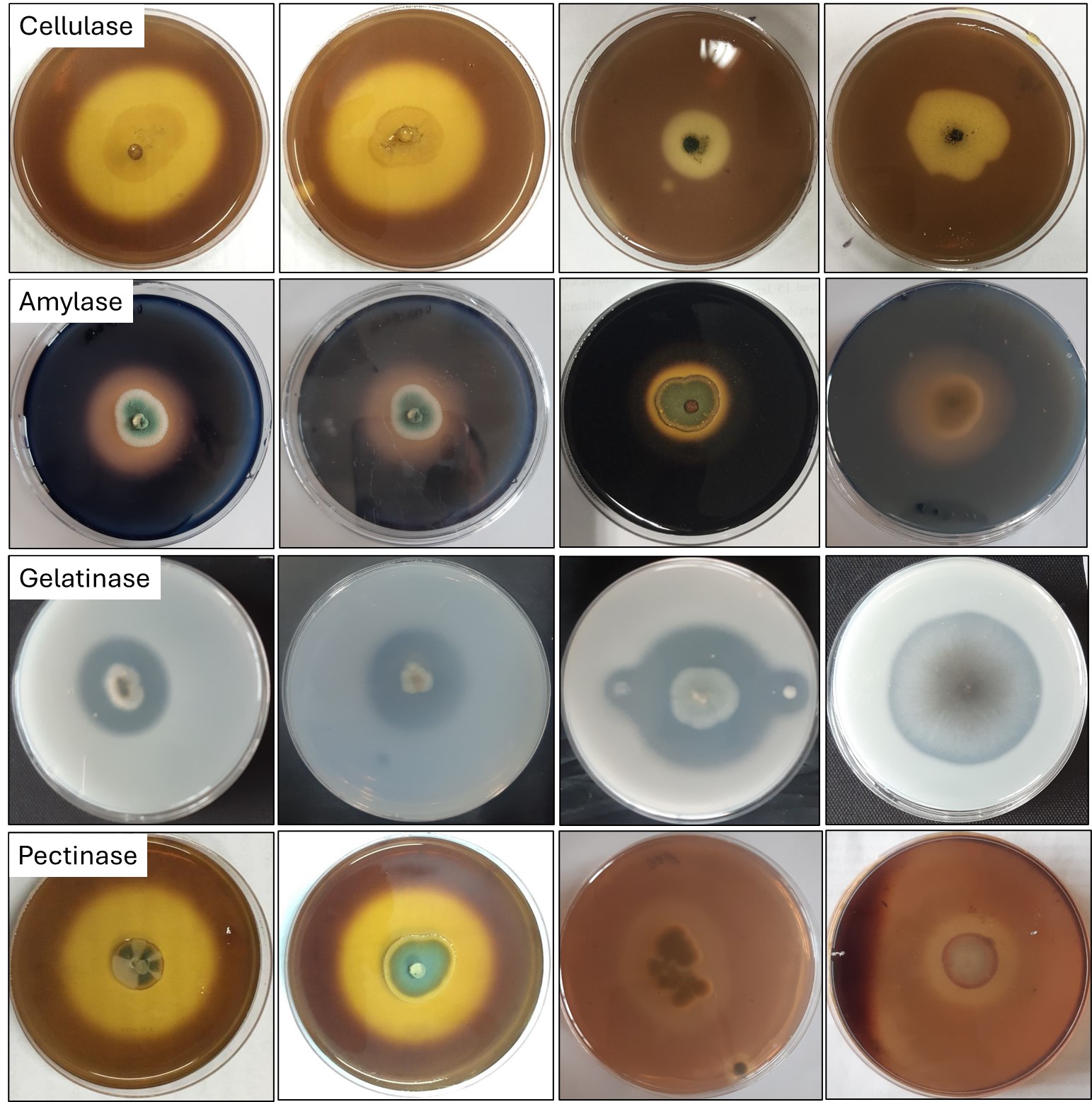


Fig. S3. Some representative plates for different enzymatic activities show varied clear zones.


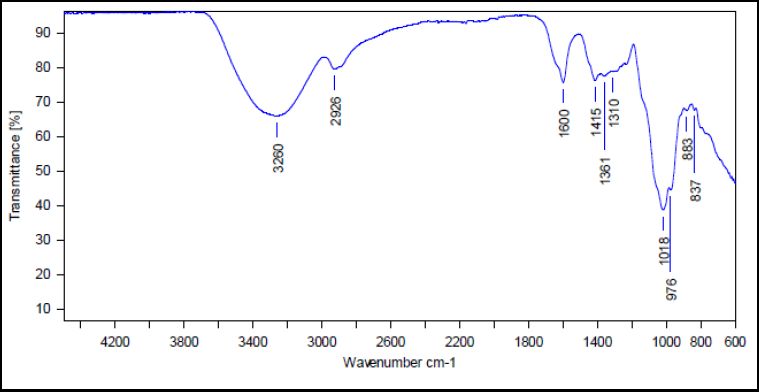


**Fig. S4.** ART-FTIR analysis of ink medium.


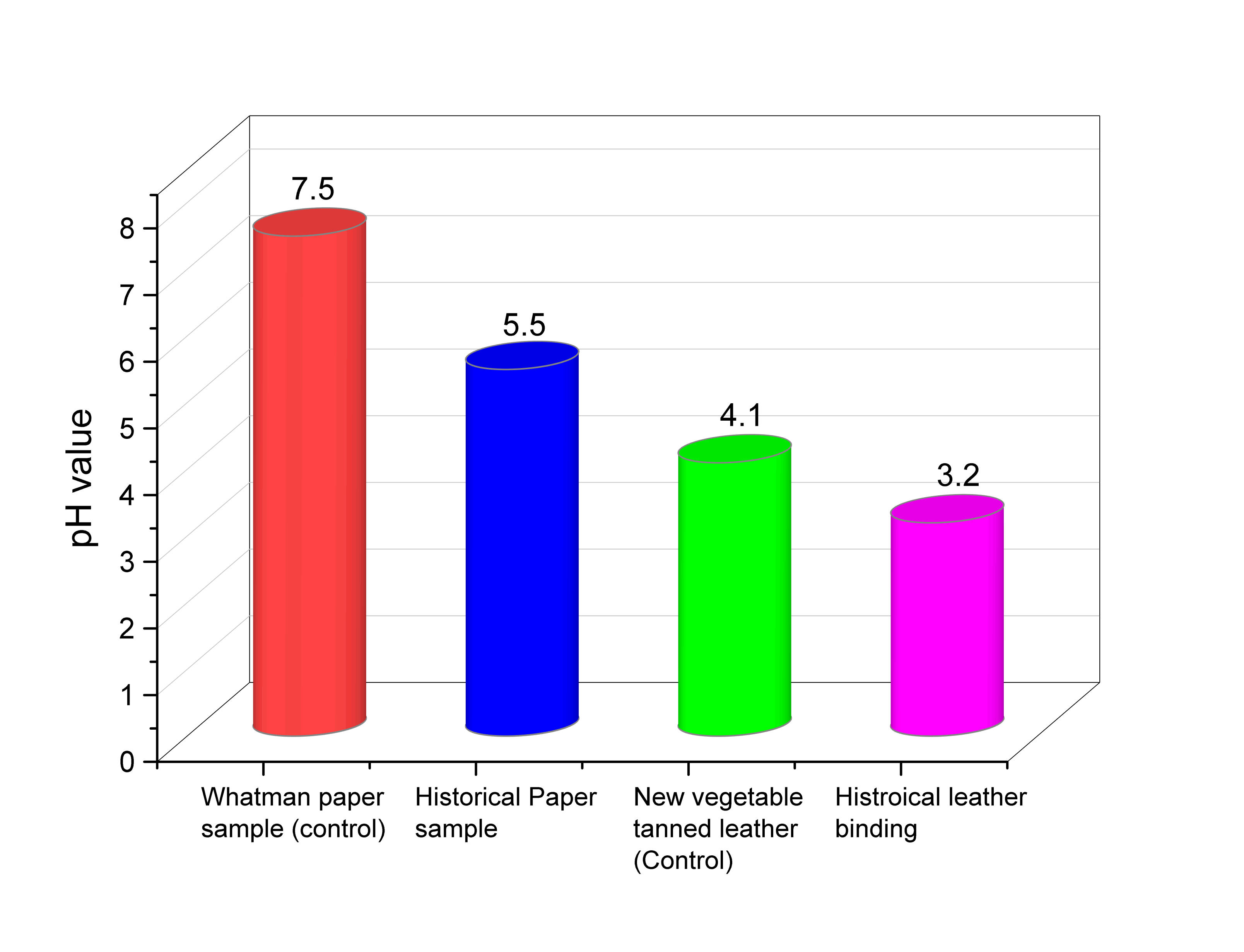


**Fig. S5.** The pH values of the historical paper and leather binding compared to control.

**
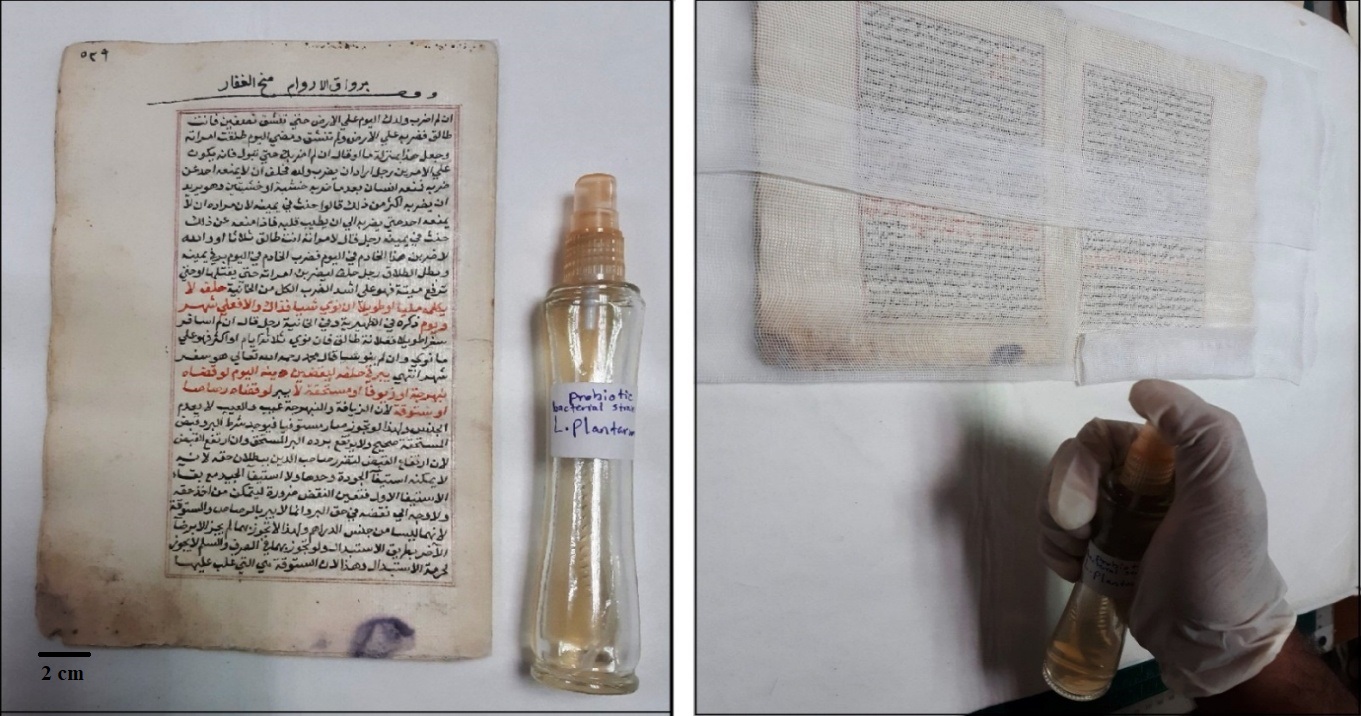
**

**Fig. S6.** Disinfection of the paper sheets of the studied manuscript
